# The effect of vagal nerve stimulation treatment on autonomic nervous system in patients with refractory epilepsy

**DOI:** 10.3389/fneur.2025.1566497

**Published:** 2025-03-28

**Authors:** Fatma Genç, Meltem Korucuk, Firdevs Ezgi Uçan Tokuç

**Affiliations:** Department of Neurology, Antalya Provincial Health Directorate, Antalya Training and Research Hospital, Antalya, Türkiye

**Keywords:** drug-resistant epilepsy, vagal nerve stimulation, sympathetic skin response, R-R interval variability, autonomic nervous system

## Abstract

**Introduction:**

Vagal nerve stimulation (VNS) is a treatment that can be used in drug-resistant epilepsy (DRE) patients who are not suitable for resective surgery. Effects of VNS on the autonomic system are controversial. In our study, we examined SSR and R-R interval variability (RR-IV) to evaluate autonomic functions in patients with refractory epilepsy treated with and without VNS and healthy volunteers.

**Methods:**

Our study included 41 healthy volunteers without any disease or drug administration, 38 DRE patients without VNS, and 38 DRE patients with VNS. Electrophysiological tests of sympathetic skin response (SSR) and RR interval variability (RR-IV) analysis were performed.

**Results:**

While no statistically significant difference was observed between the SSR latencies and amplitudes of the DRE group with VNS and the DRE group without VNS, when the SSR latencies of the 4 extremities of the DRE groups with and without VNS and the control group were compared, it was observed that both groups had statistically significantly longer SSR latencies in all extremities compared to the control group. A statistically significant difference was observed between the DRE with VNS group and the control group and RR-IV was lower in the DRE with VNS group

**Conclusion:**

In conclusion, our study is one of the rare studies investigating the effects of VNS on the sympathetic system in patients with refractory epilepsy. According to the SSR and RR-IV results in our study, there was no evidence that VNS caused sympathetic dysfunction. However, VNS may cause a shift in cardiac sympathovagal balance toward sympathetic dominance.

## 1 Introduction

Drug-resistant epilepsy (DRE) is defined as failure to achieve seizure freedom despite 2 attempts of anti-seizure medication (ASM; monotherapy and/or polytherapy) used for an appropriate duration at appropriately selected and tolerated doses (1). DRE is associated with poor outcomes both socially and individually, including death in the short and long term. Resective surgery should be considered in the treatment of DRE, if possible. In patients who are not suitable for resective surgery, neuromodulation methods such as vagal nerve stimulation (VNS) and deep brain stimulation can be utilized in addition to ASM drug treatment (2). Although the mechanism of action of VNS applied by placing a spiral electrode around the carotid sheath and a signal stimulator on the upper chest wall has not been fully elucidated, it is hypothesized that action potentials generated after stimulation project to the locus coeruleus, hypothalamus, amygdala, and thalamus via nucleus tractus solitarius stimulation and have therapeutic effect (3). Previous studies show that about 50 to 60% of patients achieve about 50% reduction in seizure frequency after 3 years of treatment after VNS administration and response rates increase over time, possibly related to neuromodulatory effects with ongoing stimulation (4, 5).

Previous studies have shown that VNS induces interictal cardiac electrical instability (elevated T wave alternans) in patients with drug-resistant epilepsy (6). Considering that T-wave alternans increase with sympathetic activation, it is believed that these findings may be related to the reduction of sympathetic tone by VNS (7). In a study conducted by Clancy et al. in a healthy population, it was reported that muscle sympathetic nerve activity was suppressed by non-invasive stimulation of the auricular branch of the vagal nerve (8). Sympathetic skin response (SSR) was examined by Yuan et al. to measure sympathetic activity in DRE patients treated with VNS, and a lower SSR was obtained in patients with VNS compared to those without VNS (9).

In our study, we examined SSR and R-R interval variability (RR-IV) to evaluate autonomic functions in patients with refractory epilepsy treated with and without VNS and healthy volunteers. We also aimed to investigate the effects of VNS parameters on SSR.

## 2 Materials and methods

### 2.1 Study population

This study was approved by the ethics committee of Antalya Training and Research Hospital (05/12/2024-19/10).

Our study included 41 healthy volunteers without any disease or drug administration, 38 DRE patients who met the definition of DRE according to ILAE diagnostic criteria, and 38 DRE patients with VNS (model 103 neurocybernetic prosthesis; Cyberonics, Pulse Generator, Houston, Texos, United States of America) who were found unsuitable for surgical treatment after long-term video EEG monitoring (1).

Patients with any disease other than epilepsy or using medications other than anti-seizure medications were excluded from the study. Patients who smoked were also excluded because it may affect the SSR.

### 2.2 Electrophysiologic tests evaluating the autonomic nervous system

All electrophysiologic examinations performed in the EMG laboratory of the Department of Neurology, Antalya Training and Research Hospital were performed with a Natus Keypoint Focus (Galway, Ireland) EMG device. Patients were asked to refrain from heavy physical activity, caffeinated beverages and nicotine use for at least 24 h before enrollment.

Standard superficial disc recording electrodes were utilized for sympathetic skin response recordings. The active recording electrode was placed on the bilateral palm and the planta, and the reference electrodes were placed on the dorsum of the hand and the dorsum of the foot. A ground electrode was placed on the right wrist. Recordings were performed with a lower frequency filter of 0.5 Hz and an upper frequency filter of 2 kHz. Sensitivity was determined as 1 mV/division and sweep rate as 1 s/division. Recordings were realized between 14:00 and 16:00 during the day. Attention was paid to ensure that the room where the recordings were made was well ventilated and quiet. The examinations were performed while the participants were lying in a comfortable supine position on a stretcher, and the skin temperature was kept above 32°C. Electrical stimulation (for 0.2 ms and 30–50 mA intensity) was applied to the right median nerve. “SSR Latency (ms) was recorded as the time required to reach the onset of the first deflection of the wave and SSR amplitude (mV) as the peak-to-peak distance of the wave.” Participants who were stimulated at a maximum intensity of 50 mA and no response was obtained were considered non-responders.

For RR-IV measurement, the active superficial disc recording electrode was placed on the left 5th rib and the reference electrode on the sternum; the ground electrode was placed on the right wrist. Recordings were performed with a lower frequency filter of 5 Hz and an upper frequency filter of 100 Hz. Sensitivity was set to 0.3–0.5 mV/division and sweep rate to 500 msec/division. In the first phase, QRS complexes were recorded for 60 s while participants were breathing normally in the supine position on a stretcher. In the second phase, participants were asked to inhale as deeply as possible and recorded for 60 s. RR-IV variability was calculated both at rest and during deep breathing (HV).

The following formula was utilized to calculate the percentage of RR-IV (RR-IV%): RR-IV% = (longest RR–shortest RR) × 100/mean of RR values (the difference between the shortest and longest RR intervals over 1 min is given as a percentage of the mean of all maximal and minimal peaks).

### 2.3 Statistical analysis

The data were analyzed with IBM SPSS V23. The conformity of the data to a normal distribution was evaluated with the Kolmogorov-Smirnov Test. Kruskal-Wallis H test was utilized to analyze the non-normally distributed data in groups of three or more, and multiple comparisons were made with Dunn's test. Mann Whitney U test was used to compare the data that did not conform to a normal distribution in paired groups. Independent samples *T*-test was used to compare the data that conformed to a normal distribution in paired groups. Pearson Chi-Square Test was used to evaluate the relationship between categorical data. Mean ± standard deviation and median (minimum-maximum) were used to represent quantitative data. Frequency and percentage were utilized in the presentation of categorical data. The significance level was taken as *p* < 0.05.

## 3 Results

The DRE with VNS group consisted of 38 patients (19 males, median age 33.2 ± 7.6), the DRE without VNS group consisted of 38 patients (22 males, median age 35.8 ± 7.6) and the control group consisted of 41 healthy volunteers (15 males, median age 34.1 ± 7.9). There was no significant difference between the patient and control groups in terms of age (*p* = 0.330) or sex (*p* = 0.158). The mean disease duration of DRE patients with VNS was 26.3 ± 8.5 years, and the mean duration of VNS treatment was 4 ± 2.7 years (range from 1 to 9 years). The mean duration of disease in the DRE without VNS group was 23 ± 9.7 years. Sympathetic skin response could not be obtained in 9 (23.7%) patients in the DRE with VNS group and in 10 (26.3%) patients in the DRE without VNS group. The demographic data of the participants and their SSR, RR-IV status during rest and HV are presented in Table 1.

**Table 1:** Descriptive statistics.

	**DRE with VNS**	**DRE without VNS**	**Control**	**Test statistics**	***p***
Age (years, mean ± s.d)	33.2 ± 7.6	35.8 ± 7.6	34.1 ± 7.9	2.216	0.330^*x*^
Sex (male, %)	19 (50)	22 (57.9)	15 (36.6)	3.692	0.158^*x*^
Duration of illness (years)	26.3 ± 8.5	23 ± 9.7	-	1.584	0.118^*y*^
Age of disease onset (years)	6 (0–24)	12 (1–34)	-	394.500	**< 0.001** ^*^
Number of ASM	4 (2–6)	4 (3–6)	-	981.500	**0.005** ^z*^
Seizure frequency (per month)	3.5 (0–45)	1 (0–60)	-	1068.000	**< 0.001** ^z*^
VNS duration (years)	4 ± 2.7		-		
SSR (no response)	9 (23.7)^a^	10 (26.3)^a^	0 (0)^b^	12.334	**0.002** ^x*^
Rest RR-IV (no response)	10 (26.3)^a^	8 (21.1)^a^	0 (0)^b^	11.880	**0.003** ^x*^
HV RR-IV (no response)	16 (42.1)^a^	10 (26.3)^a^	0 (0)^b^	20.774	**< 0.001** ^x*^

^*x*^Kruskal Wallis H Test; ^*y*^Independent samples T Test; ^*z*^Mann Whitney U Test; ^*a*−*b*^no difference between groups with the same letter; ^*^p < 0.05 indicates a significant difference between the groups. Median (minimum-maximum), Mean ± Standard deviation.

DRE, Drug-resistant epilepsy; VNS, Vagal nerve stimulation; ASM, Anti-seizure medication; SSR, Sympathetic skin response; RR-IV, R-R interval variability; HV, Hyperventilation.

**Figure 1:**
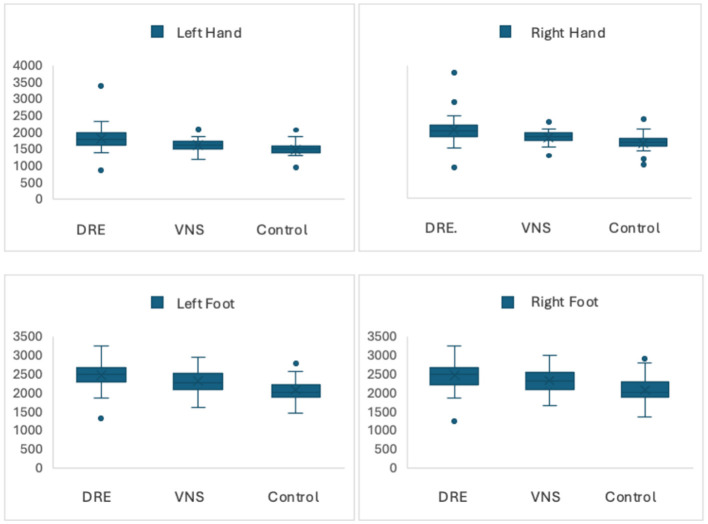
SSR latencies by groups.

**Figure 2:**
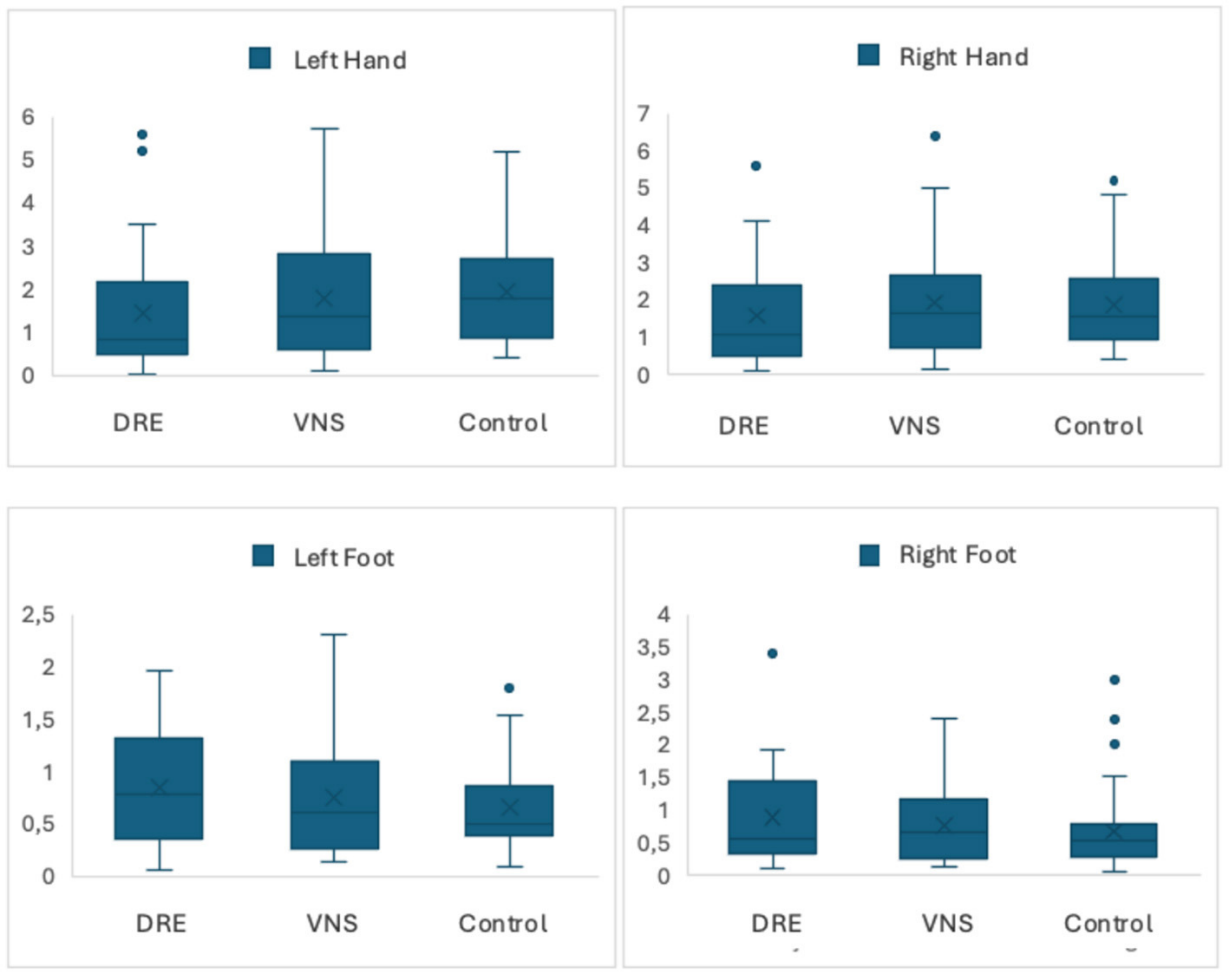
SSR amplitudes by groups.

While no statistically significant difference was observed between the SSR latencies and amplitudes of the DRE group with VNS and the DRE group without VNS, when the SSR latencies of the 4 extremities of the DRE groups with and without VNS and the control group were compared, it was observed that both groups had statistically significantly longer SSR latencies in all extremities compared to the control group (*p* < 0.001, < 0.001, < 0.001, < 0.001; Table 2; Figures 1, 2).

**Table 2:** Comparison of quantitative variables according to groups.

	**DRE with VNS**	**DRE without VNS**	**Control**	**Test Statistics**	***p***
Left hand latency (ms)	1608.5 (1,171–2,066)^*a*^	1780.5 (844–3,391)^*a*^	1,490 (947–2,061)^*b*^	26.550	**< 0.001** ^x*^
Left hand amplitude (mV)	1.37 (0.087–5.7)	0.845 (0.039–5.6)	1.79 (0.39–5.2)	4.146	0.126^*x*^
Right hand latency (ms)	1,632 (1,131–2,005)^*a*^	1,772 (823–3,300)^*a*^	1,473 (903–2,080)^*b*^	28.723	**< 0.001** ^x*^
Right hand amplitude (mV)	1.63 (0.15–6.4)	1.07 (0.09–5.6)	1.56 (0.42–5.2)	2.544	0.280^*x*^
Left foot latency (ms)	2267.5 (1,603–2,940)^*a*^	2,474 (1,321–3,227)^*a*^	2,016 (1,337–2,840)^*b*^	18.433	**< 0.001** ^x*^
Left foot amplitude (mV)	0.6 (0.14–2.3)	0.775 (0.064–1.96)	0.5 (0.083–1.79)	1.391	0.499^*x*^
Right foot latency (ms)	2,307 (1,660–2,977)^*a*^	2,480 (1,248–3,251)^*a*^	2,006 (1,353–2,897)^*b*^	19.350	**< 0.001** ^x*^
Right foot amplitude (mV)	0.66 (0.12–2.4)	0.555 (0.11–3.4)	0.53 (0.05–3)	1.100	0.577^*x*^

^*x*^Kruskal Wallis H Test; ^*a*−*b*^no difference between groups with the same letter; ^*^p < 0.05 indicates a significant difference between the groups. Median (minimum-maximum).

DRE, Drug-resistant epilepsy; VNS, Vagal nerve stimulation.

When the factors affecting non-response in SSR assessment were analyzed, an increase in seizure frequency led to an increased risk of non-responders (*p* = 0.029). No association was observed between ASM, disease duration, age at disease onset and SSR (*p* > 0.05; Table 3).

**Table 3:** Binary logistic results for SSR status.

	**SSR**		**Univariate**		**Multiple**	
	**No response**	**Response**	**OR (%95 CI)**	**p**	**OR (%95 CI)**	**p**
Seizure frequency (per month)	11.53 ± 17.5	3.95 ± 7.21	0.95 (0.9–0.99)	**0.024** ^*^	0.95 (0.9–0.99)	**0.029** ^*^
Number of ASM	4.53 ± 1.26	3.98 ± 0.92	0.60 (0.36–1)	0.051	0.59 (0.35–1.01)	0.056
Duration of illness (years)	24.53 ± 8.62	24.72 ± 9.57	1 (0.95–1.06)	0.937	1 (0.93–1.08)	0.988
Age of disease onset (years)	9.47 ± 8.87	10.11 ± 7.39	1.01 (0.94–1.08)	0.756	0.99 (0.92–1.07)	0.857

^*^p < 0.05 indicates a significant difference between the groups. OR, Odds ratio; SSR, Sympathetic skin response; ASM, Anti-seizure medication.

There was a statistical difference between the resting RR-IV values between the groups (*p* = 0.004). A statistically significant difference was observed between the DRE with VNS group and the control group and RR-IV was lower in the DRE with VNS group. There was no statistically significant difference between the DRE with VNS group and the DRE without VNS group (*p* > 0.05; Table 4).

**Table 4:** Comparison of quantitative variables according to groups.

	**DRE with VNS**	**DRE without VNS**	**Control**	**Test statistics**	***p***
Rest RR-IV	17.55 (6.5–27.7)^*a*^	18.8 (5.6–36.2)^*ab*^	23.2 (7.5–57)^*b*^	10.902	**0.004** ^x*^
Hv RR-IV	25.65 (13.3–50.9)^*a*^	29.05 (8.1–58.5)^*a*^	42.6 (22.2–72.8)^*b*^	31.415	**< 0.001** ^x*^

^*x*^Kruskal Wallis H Test; ^*a*−*b*^no difference between groups with the same letter; ^*^p < 0.05 indicates a significant difference between the groups. DRE, Drug-resistant epilepsy; VNS, Vagal nerve stimulation; RR-IV, R-R interval variability; HV, Hyperventilation.

According to the groups, there was a statistically significant difference between the RR-IV values in the HV period (*p* < 0.001) and it was statistically significantly lower in the DRE with VNS group compared to the control group, while no statistical difference was observed between the DRE with VNS group and DRE without VNS group (*p* > 0.05; Table 4).

There was no statistically significant difference between SSR non-response and VNS parameters (Table 5). In addition, no statistically significant difference was observed when the variables of RRIV at resting and HV state and VNS parameters were examined (*p* > 0.05; Tables 6, 7).

**Table 5:** Comparison of SSR status and quantitative variables.

	**No response**	**Response**	**Total**	**Test statistics**	***p***
VNS duration (years)	3 (1–9)	2 (1–9)	2.5 (1–9)	147.000	0.574^*x*^
VNS output (mA)	2.167 ± 0.375	1.922 ± 0.672	1.98 ± 0.619	1.383	0.179^*y*^
Signal frequency (Hz)	30 (25–30)	30 (20–30)	30 (20–30)	130.000	1.000^*x*^
Pulse width (μs)	500 (500–500)	500 (130–500)	500 (130–500)	171.000	0.064^*x*^
Signal on time (seconds)	30 (30–60)	30 (21–60)	30 (21–60)	165.500	0.062^*x*^
Signal off time (minutes)	1.8 (1.8–5)	3 (1.8–5)	3 (1.8–5)	80.500	0.072^*x*^

^*x*^Mann Whitney U Test; ^*y*^Independent Samples T Test; Median (minimum-maximum), Mean ± Standard deviation.

VNS, Vagal nerve stimulation.

**Table 6:** Comparison of resting RR-IV with quantitative variables.

	**Impaired**	**Normal**	**Total**	**Test statistics**	***p***
VNS duration	5 (1–9)	2 (1–9)	2.5 (1–9)	149.500	0.760^*x*^
VNS output (mA)	2 (1.25–2.75)	2.25 (0.75–3)	2.25 (0.75–3)	137.000	0.933^*x*^
Signal frequency (Hz)	30 (25–30)	30 (20–30)	30 (20–30)	141.500	0.950^*x*^
Pulse width (μs)	500 (250–500)	500 (130–500)	500 (130–500)	167.500	0.228^*x*^
Signal on time (seconds)	30 (30–30)	30 (21–60)	30 (21–60)	140.000	1.000^*x*^
Signal off time (minutes)	4 (1.8–5)	3 (1.8–5)	3 (1.8–5)	177.500	0.193^*x*^

^*x*^Mann Whitney U Test; Median (minimum-maximum).

VNS, Vagal nerve stimulation.

**Table 7:** Comparison of HV RR-IV with quantitative variables.

	**Impaired**	**Normal**	**Total**	**Test statistics**	***p***
VNS duration	2.5 (1–9)	3 (1–9)	2.5 (1–9)	152.000	0.477^*x*^
VNS output (mA)	1.891 ± 0.677	2.046 ± 0.581	1.98 ± 0.619	−0.757	0.454^*y*^
Signal frequency (Hz)	30 (25–30)	30 (20–30)	30 (20–30)	171.000	0.803^*x*^
Pulse width (μs)	500 (130–500)	500 (130–500)	500 (130–500)	191.000	0.564^*x*^
Signal on time (seconds)	30 (21–30)	30 (21–60)	30 (21–60)	141.500	0.113^*x*^
Signal off time (minutes)	3 (1.8–5)	3 (1.8–5)	3 (1.8–5)	201.000	0.442^*x*^

^*x*^Mann Whitney U Test; ^*y*^Independent Samples T Test; Median (minimum-maximum), Mean ± Standard deviation.

VNS, Vagal nerve stimulation.

## 4 Discussion

The primary result of our study was that DRE patients with and without VNS had prolonged SSR latencies compared to the control group, while there was no significant difference between the SSR latencies and amplitudes in DRE patients with and without VNS.

Several previous studies have suggested that VNS administered in healthy volunteers and patients with Long QT syndrome decreases sympathetic response (8, 10). When we search for studies investigating the effects of VNS on the sympathetic system in epilepsy patients, only one study in the literature draws attention. Yuan et al. performed SSR recording in 6 DRE patients with VNS and 20 DRE patients without VNS, and SSR amplitudes were found to be significantly lower in DRE patients with VNS compared to patients without VNS (9). In contrast to these studies, our study did not reveal any evidence that VNS affects the sympathetic system in patients with refractory epilepsy according to the results of SSR.

Numerous studies have previously evaluated the functions of the autonomic nervous system during ictal or interictal periods in epileptic patients. In these studies, changes in SSR amplitude and latencies were observed. The general opinion is that sympathetic dysfunction develops in the ictal and interictal periods in patients with epilepsy (11–14). Sympathetic skin responses are considered to receive suprasegmental excitatory inputs from the cerebral cortex and suprasegmental inhibitory inputs from the striatum and reflect the activity of the posterior hypothalamus and brainstem reticular formation. It has been suggested that SSR latency reflects the conduction of the efferent sudomotor pathway and postganglionic unmyelinated C fibers (15, 16). It is hypothesized that sympathetic nervous system dysfunction is more objectively reflected by SSR latency values (17).

In addition, another factor that may affect SSR in epilepsy patients is anti-seizure medications. Especially Na channel blocking ASMs have been suggested to have effects on cardiac autonomic function. While some studies have argued that levetiracetam activates the sympathetic system, some studies have claimed that it has no effect on the autonomic system (18–21). The results obtained in our study show that refractory epilepsy is a disease that causes sympathetic dysfunction. We also observed that high seizure frequency was the only factor affecting SDR. These data are similar to studies showing that sympathetic dysfunction develops in the ictal and interictal periods in epilepsy patients. Studies have shown that each seizure causes sudden and transient impairments in autonomic functions and seizure repetitions lead to long-term abnormalities in autonomic systems (11–14).

The secondary result of our study is that in individuals with VNS, RR-IV values were low both at rest and in HV. RR-IV is under parasympathetic system control, and its decrease or disappearance reflects parasympathetic dysfunction (22). In previous studies, RR-IV values were found to be lower in individuals with epilepsy compared to healthy individuals, and it has been suggested that epilepsy may cause cardiac parasympathetic dysfunction (11, 17). Many studies have been conducted on the effects of VNS on HRV (23–25). Although the results of these studies are contradictory, the results of 2 studies are remarkable: In the study conducted by Jansen et al., it was observed that VNS caused sympathetic shift in cardiac sympathovagal balance (26). Galli et al. detected a decrease in nocturnal vagal activity after long-term VNS administration (27). These results are consistent with our RR-IV data observed in individuals with VNS. Although the mechanism of action of VNS is not fully understood, prolonged stimulation of the vagus nerve in rats has shown increased activity of serotonin and noradrenaline neurons in the dorsal raphe nucleus and locus coeruleus. Locus coeruleus neurons project to the hippocampus and prefrontal cortex and are the source of hippocampal and cortical noradrenaline (26, 28). Based on this, the decrease in RR-IV in individuals with VNS observed in our study (considering that RR-IV is an indicator of vagal activity) may be explained as VNS may cause an increase in cardiac sympathetic activity by causing an increase in the activity of noradrenaline neurons, but this should be investigated in more detail (29).

Our study has some limitations. First of all, the small sample size may have affected our results. In addition, our data could have been supported with additional tests (Valsalva test, quantitative sudomotor axon reflex test, etc.) for autonomic nervous system evaluation. Another limitation of our study is that the relationship between seizure types and the time elapsed since the last seizure and SDR was not examined. However, seizure anamnesis obtained from patients and/or their relatives was not included in our study considering that it may be misleading. Again, since we did not have data on the time elapsed since the last seizure, the relationship with SDR could not be examined.

In conclusion, our study is one of the rare studies investigating the effects of VNS on the sympathetic system in patients with refractory epilepsy. According to the SSR and RR-IV results in our study, there was no evidence that VNS caused sympathetic dysfunction. However, VNS may cause a shift in cardiac sympathovagal balance toward sympathetic dominance, and further studies are needed.

## Data Availability

The original contributions presented in the study are included in the article/supplementary material, further inquiries can be directed to the corresponding author.
